# The incidence, mortality and renal outcomes of acute kidney injury in patients with suspected infection at the emergency department

**DOI:** 10.1371/journal.pone.0260942

**Published:** 2021-12-08

**Authors:** Meriem Khairoun, Jan Willem Uffen, Gurbey Ocak, Romy Koopsen, Saskia Haitjema, Jan Jelrik Oosterheert, Karin Kaasjager

**Affiliations:** 1 Department of Nephrology and Hypertension, University Medical Center Utrecht, Utrecht, Netherlands; 2 Department of Acute Internal Medicine, University Medical Center Utrecht, Utrecht, Netherlands; 3 Department of Internal Medicine and Nephrology, Sint Antonius Hospital, Nieuwegein, Netherlands; 4 Department of Laboratory of Clinical Chemistry/Haematology, University Medical Center Utrecht, Utrecht, Netherlands; Jouf University, Kingdom of Saudi Arabia, SAUDI ARABIA

## Abstract

**Background:**

Acute kidney injury (AKI) is a major health problem associated with considerable mortality and morbidity. Studies on clinical outcomes and mortality of AKI in the emergency department are scarce. The aim of this study is to assess incidence, mortality and renal outcomes after AKI in patients with suspected infection at the emergency department.

**Methods:**

We used data from the SPACE-cohort (SePsis in the ACutely ill patients in the Emergency department), which included consecutive patients that presented to the emergency department of the internal medicine with suspected infection. Hazard ratios (HR) were assessed using Cox regression to investigate the association between AKI, 30-days mortality and renal function decline up to 1 year after AKI. Survival in patients with and without AKI was assessed using Kaplan-Meier analyses.

**Results:**

Of the 3105 patients in the SPACE-cohort, we included 1716 patients who fulfilled the inclusion criteria. Of these patients, 10.8% had an AKI episode. Mortality was 12.4% for the AKI group and 4.2% for the non-AKI patients. The adjusted HR for all-cause mortality at 30-days in AKI patients was 2.8 (95% CI 1.7–4.8). Moreover, the cumulative incidence of renal function decline was 69.8% for AKI patients and 39.3% for non-AKI patients. Patients with an episode of AKI had higher risk of developing renal function decline (adjusted HR 3.3, 95% CI 2.4–4.5) at one year after initial AKI-episode at the emergency department.

**Conclusion:**

Acute kidney injury is common in patients with suspected infection in the emergency department and is significantly associated with 30-days mortality and renal function decline one year after AKI.

## Introduction

Acute kidney injury (AKI) is a major health problem associated with considerable mortality and morbidity. Globally, AKI is associated with increased length of hospital stay, need for complex interventions (e.g., renal replacement therapy (RRT)) and use of critical care services, leading to a high financial burden [1–3]. Depending on what definition is used, the incidence of AKI ranges between 5–30% in hospitalized patients and 40–65% in patients at the intensive care unit (ICU) [4–6].

Although the etiology of AKI is multifactorial, sepsis of any cause, including infection is the most common cause of AKI in critically ill patients [7–9]. Sepsis as cause of AKI in adult intensive care units has been reported in about 50–70% [7, 10]. In addition, depending on AKI severity, 90-day mortality rates ranged from 16–50% in septic patients with AKI at the ICU [11]. Moreover, in uncomplicated community acquired pneumonia the incidence of AKI was high and associated with lower survival [12].

Given the complex pathophysiology of AKI and the various comorbidities of affected patients, recognition and management of AKI remains complicated [13, 14]. Inadequate recognition and treatment of AKI increases the susceptibility to chronic kidney disease (CKD) and end-stage renal disease (ESRD) with requirement for RRT and an increased mortality risk [15]. Moreover, recent studied have found that AKI was independently associated with an increased risk for a subsequent infection in different patient populations with and without sepsis and infection, up to 1 year after AKI [16].

The epidemiology of AKI in hospitalized and critically ill patients in the ICU with severe infection and sepsis has been well described, however data on mortality and clinical outcomes of AKI in the emergency department setting in patients with suspected infection are scarce [8, 17]. Since emergency departments are frequently the first site of care for patients with AKI, early identification may help to improve standards of care and patient outcomes [18, 19]. Therefore, we investigated the incidence, 30-days mortality and renal outcomes after AKI up to one year after initial AKI-episode in a unique cohort of patients with suspected infection at the emergency department of a tertiary care center.

## Material and methods

### Study design and setting

**Fig 1:**
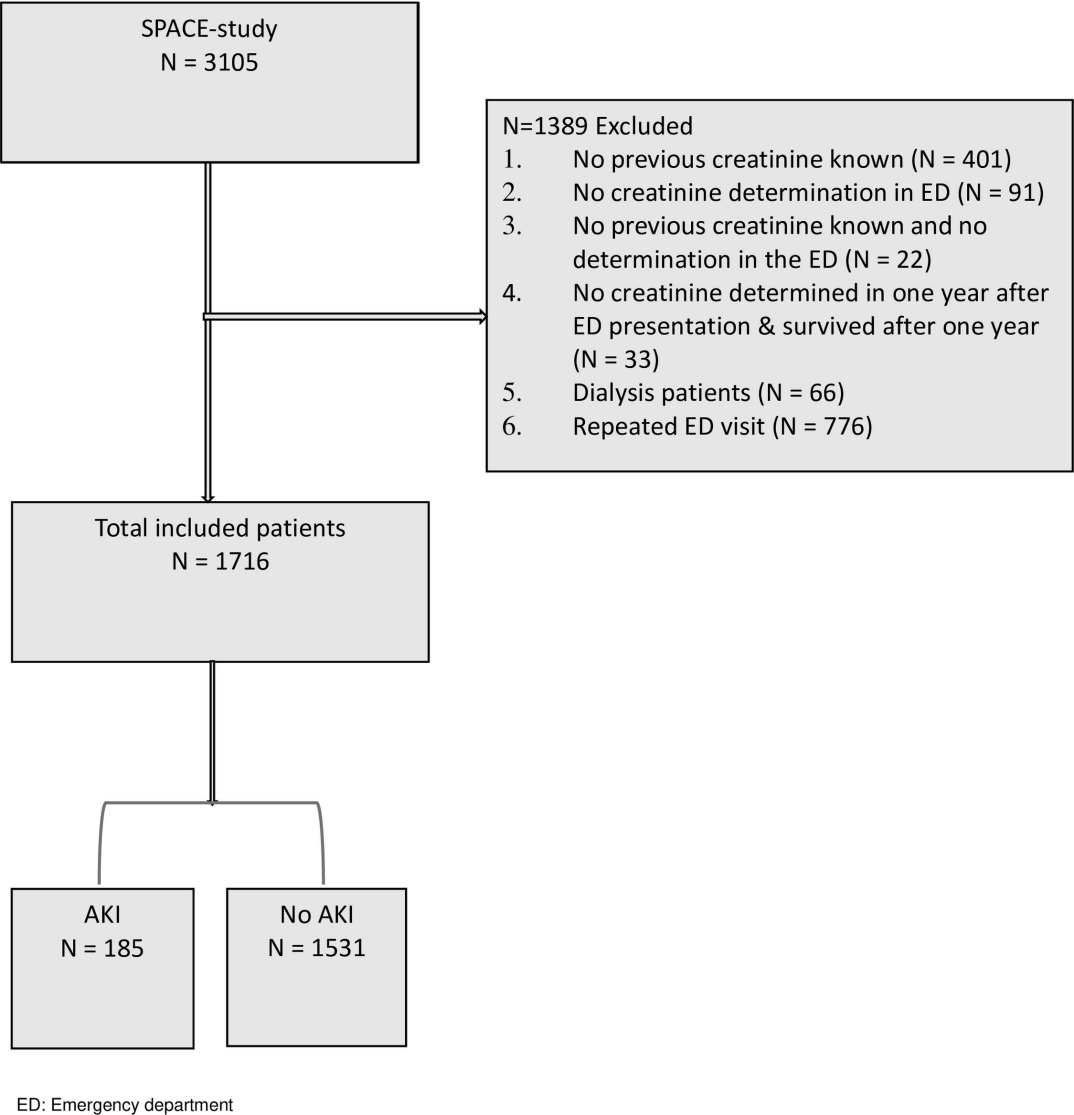
Flowchart of patients included in the analyses.

For the current study we used data from the SPACE-cohort (SePsis in the ACutely ill patients in the Emergency department) [20]. In short, this cohort consists of all consecutive patients presenting at the emergency department of the internal medicine with suspected infection in the University Medical Center Utrecht (UMCU). Analysis was performed on data collected prospectively from September 13rd 2016 until October 11th 2018 and included 1716 patients (Fig 1). The UMCU is one of the largest tertiary care centres in the Netherlands. The SPACE-cohort and current study were reviewed and approved by the Medical Ethical Committee of the UMCU under number 16/594 and registered in the Dutch Trial Register (NTR) under number 6916. The review board waived the need for written patient consent as only pseudonymized data were included in current study.This study was conducted flowing the 1975 Helsinki declaration.

### Population and data collection

The SPACE-cohort consists of all consecutive patients who meet the following criteria: (1) ≥ 18 years or older; (2) presentation at the emergency department with the suspicion of an infection defined by the treating physician in the emergency department; (3) registration in the emergency department for the internal medicine department or its subspecialties (oncology, rheumatology, immunology, hematology, nephrology, endocrinology, gastro-enterology, infectious disease and vascular medicine). Patient data collected in this cohort consists of all clinical data upon presentation in the emergency department and, if applicable, hospital admission, laboratory data, treatment data and outcome data including mortality.

For all patients, SIRS and qSOFA and Modified Early Warning Score (MEWS) were automatically calculated in the emergency department based on the first complete available record. MEWS included blood pressure, pulse rate, respiratory rate, body temperature, consciousness. Each condition was given a score of 0–3, and the sum of the scores was calculated. A higher MEWS score indicates an increased disease severity. SIRS was considered present in case of two or more of the following criteria: heart rate ≥ 90 per minute, leukocyte count ≥ 12.0 or ≤ 4.0 (x10^9/l), respiratory rate ≥ 20 per minute or temperature ≥ 38.0 or ≤ 36.0°C. qSOFA was considered positive in case of two or more of the following criteria: respiratory rate ≥ 22 per minute, Glasgow Coma Score (GCS) ≤14 or systolic blood pressure < 100 mmHg.

Data on general patient information, vital signs, laboratory testing (including serum creatinine (SCr) and estimated glomerular filtration rate (eGFR)), immune status, medication use and mortality were automatically extracted from the Electronic Health Records.

Diagnosis at admission and diagnosis at discharge were retrieved from the emergency department record and hospital discharge letter, respectively. These diagnoses were reviewed on correctness and accuracy by a standardized independent review of the medical record by two principal investigators. This review was based on symptoms, vital signs, laboratory results, radiology and microbiology results and was standardized for most common infections.

### Definitions acute kidney injury

The diagnosis and staging of AKI in adults were defined according to the Kidney Disease Improving Global Outcomes (KDIGO) criteria [21]. Data on urinary output were not available in this SPACE-cohort and were not included in the definition of AKI. Baseline SCr was defined as the last available SCr level up to 12 months prior to presentation to the emergency department [22, 23]. The median period between baseline SCr and presentation at the emergency department was 28 days (IQR 13–73). Patients receiving RRT or without SCr value at baseline or during emergency department visit were excluded. Kidney transplant recipients were included in the current study and considered as immunocompromised for the analyses. Follow-up SCr and eGFR data were obtained of all patients if available, up to 12 months after initial AKI episode.

### Outcomes

The primary outcomes of this study were 30-days all-cause mortality and renal function decline after AKI up to 1 year in patients with suspected infection at the emergency department, up to one year after presentation. Secondary outcomes were in-hospital mortality and association with AKI. Decline of renal function after AKI episode at emergency department visit was defined as SCr ≥30% above baseline [24]. For assessment of renal function after discharge, we included the latest available SCr value up to 12 months after initial presentation. Data on comorbidity was quantified using the Charlson Comorbidity Index (CCI).

### Statistical analyses

Data are presented as median and interquartile range and compared using a Man-Whitney U tests. Categorical variables are presented as frequencies and percentages and compared using Chi-square tests. Survival curves of mortality were determined with the Kaplan–Meier method for the AKI and non-AKI group. Significant differences in survival curves were analyzed using Log-rank test. We calculated hazard ratios (HRs) with 95% confidence intervals (CIs) using Cox regression analysis to investigate the relationship between AKI and all‐cause mortality and renal function decline. In addition, we adjusted HRs for potential confounders including age, gender, Charlson Comorbidity Index (including CKD), immune status, smoking status, medication use (diuretics, proton-pump inhibitors, non-steroidal anti-inflammatory drugs (NSAIDs) and angiotensin converting enzyme inhibitors (ACEi)), disease severity, diagnosis in the emergency department. A p-value <0.05 was considered statistically significant. Analyses were performed using SPSS-software version 25.0.

## Results

### Baseline characteristics

Of the 3105 patient in the SPACE-cohort, we included 1716 patients for this analysis after exclusion of the following patients from analyses: 401 patients had no baseline SCr, 91 patients had no SCr measurement at emergency department presentation, 22 patients missed both baseline and emergency department admission SCr value, 33 patients had no SCr determined in one year after emergency department presentation and survived after one year, 66 patients had renal replacement therapy at the time of emergency department visit and 776 patients had a repeated emergency department visit during the study period (Fig 1). The median age of the patients was 62 years (IQR 48–70) and 52.9% of the patients were male. The most common causes of infection at presentation of the emergency department were lower respiratory tract infections (20.6%), viral respiratory infections (17.9%), gastro-intestinal infections (16.3%), urinary tract infections (15.7%), and skin infections (6.6%). The most common comorbidities were hypertension (31.5%), chronic kidney disease (23.7%) and rheumatic diseases (20.5%) with a median Charlson Comorbidity Index of 4 (IQR 2–7). Of the included patients, 37.9% had an immunocompromised status (including kidney transplant recipients). Median baseline SCr at admission was 83 (IQR 65–123) umol/L (Table 1).

**Table 1:** Baseline characteristics.

	Total (N = 1716)	No AKI (N = 1531)	AKI (N = 185)	p-value
**Age median (IQR)**	62 (48–70)	62 (48–70)	61 (51–69)	0.94
Male (%)	908 (52.9)	810 (52.9)	98 (53.0)	0.98
**Comorbidities**				
Hypertension (%)	540 (31.5)	476 (31.1)	64 (34.6)	0.33
Diabetes Mellitus (%)	316 (18.4)	269 (17.6)	47 (25.4)	**< 0.01**
Severe Liver disease (%)	18 (1.0)	13 (0.8)	5 (2.7)	**0.02**
Heart failure (%)	93 (5.4)	80 (5.2)	13 (7.0)	0.31
Myocardial infarction (%)	148 (8.6)	132 (8.6)	16 (8.6)	0.99
Peripheral vascular disease (%)	138 (8.0)	112 (7.3)	26 (14.1)	**< 0.01**
Cerebrovascular disease (%)	182 (10.6)	151 (9.9)	31 (16.8)	**< 0.01**
Kidney Transplant (%)	186 (10.8)	158 (10.3)	28 (15.1)	0.05
CKD (%)	407 (23.7)	346 (22.6)	61 (33.0)	**< 0.01**
Immunocompromised	651 (37.9)	576 (37.6)	75 (40.5)	0.44
**Medication**				
ACE- inhibitor	487 (28.4)	418 (27.3)	69 (37.3)	**< 0.01**
Diuretics	342 (19.9)	290 (18.9)	52 (28.1)	**< 0.01**
PPI	858 (50.0)	748 (48.9)	110 (59.5)	**< 0.01**
NSAID	98 (5.7)	82 (5.4)	16 (8.6)	0.07
**Disease severity in ED**				
MEWS	2 (1–3)	2 (0–3)	2 (1–5)	**< 0.01**
qSOFA	0 (0–1)	0 (0–1)	1 (0–1)	**< 0.01**
**Infectious diagnosis upon presentation in the ED**				
Lower respiratory tract infection	354 (20.6)	322 (21.0)	32 (17.3)	0.24
Viral respiratory tract infection	307 (17.9)	293 (19.1)	14 (7.6)	**< 0.01**
Urinary tract infection	270 (15.7)	221 (14.4)	49 (26.5)	**< 0.01**
Gastro-intestinal infection	280 (16.3)	240 (15.7)	40 (21.6)	**0.04**
Skin infection	113 (6.6)	107 (7.0)	6 (3.2)	0.05
**Stages of AKI**				
Stage 1	-	-	124 (67.0)	-
Stage 2	-	-	41 (22.2)	-
Stage 3	-	-	20 (10.8)	-
**Laboratory (IQR)**				
Baseline serum creatinine (umol/L)	76 (63–105)	76 (63–105)	74 (58–118)	0.58
Baseline eGFR CKD-EPI (ml/min)	83 (58–100)	83 (58–100)	83 (51–100)	0.75

AKI: Acute Kidney Injury, any stage; IQR: Interquartile range; ACE: Angiotensin-converting-enzyme;PPI: Proton pump-inhibitor; ED: Emergency department; MEWS: Modified Early Warnings Score; quick Sepsis Related Organ Failure Assessment; CKD: Chronic kidney disease; eGFR: Estimated glomerular filtration rate; CKD-EPI: Chronic Kidney Disease Epidemiology Collaboration.

### AKI patients at emergency department

Of the 1716 included patients, 185 (10.8%) patients had AKI at presentation at the emergency department. Most patients had AKI stage 1 (67%), followed by stage 2 (22.2%) and stage 3 (10.8%). The median age in AKI patients was 61 years (IQR 51–69) and 53.0% were male. In patients with AKI, hypertension (34.6%), diabetes mellitus (25.4%), and cerebral vascular disease (16.8%) were the most common comorbidities. The median Charlson Comorbidity Index was 5 (IQR 3–7) in the AKI patients versus 4 (IQR 2–7) in the non-AKI group. Of the 185 patients with AKI, 15.1% had a kidney transplantation. Furthermore, patients with AKI had higher disease severity scores (qSOFA 1 (IQR 0–1) and MEWS 2 (IQR 1–5)) as compared to the non-AKI patients (qSOFA 0 (IQR 0–1) and MEWS 2 (IQR 0–3)). The baseline SCr levels were not significantly different between the AKI and non-AKI group. Most common infections in the AKI patients at admission to the emergency department were a urinary tract infection (26.5%), a gastro-intestinal infection (21.6%) and a lower respiratory tract infection (17.3%).

### Mortality and AKI

**Fig 2:**
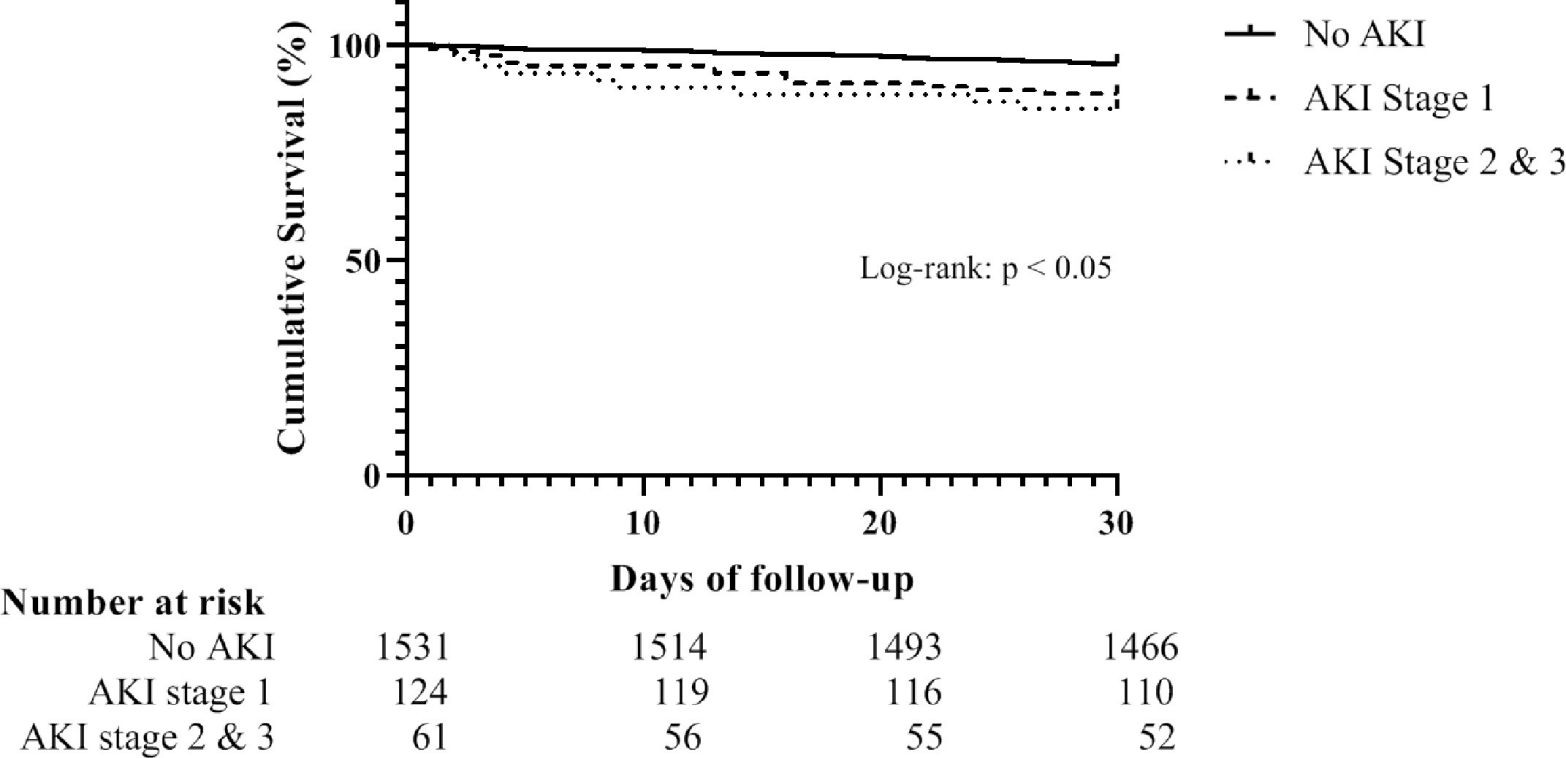
Kaplan Meijer curve for 30 days mortality comparing patients with and without acute kidney injury.

Thirty days after the initial AKI episode, 1628 patient were still alive (survivors) and 88 patients passed away (non-survivors). In Fig 2, the Kaplan-Meier survival curve for patients with AKI and without AKI is shown. The cumulative 30-days mortality was 12.4% for the AKI group and 4.2 for the non-AKI group. The mortality rate of AKI in the survivor group was 1550 per 1000 person-years versus 530 in the non-AKI group. The association between AKI and 30 days mortality remained independently associated with a HR of 2.8 (95% CI 1.7–4.8), regardless of AKI stage AKI stage 1 HR of 3.0 (95% CI 1.6–5.5) and AKI stage 2 HR 2.6 (95% CI 1.2–5.6), after adjustment for age, gender, comorbidities, immune status, smoking status, medication use, disease severity, diagnosis in the emergency department (Table 2). Consistently, adjusted in-hospital mortality was increased in patients with AKI compared to non-AKI (HR 4.0 (95% CI 2.2–7.4) (Table 3).

**Table 2:** Association between acute kidney injury and 30-days mortality.

	N	Crude HR (95%CI)	Model 1^a^ HR (95%CI)	Model 2^b^ HR (95%CI)	Model 3^c^ HR (95%CI)	Model 4^d^ HR (95% CI)
No AKI	1531	1.0 (reference)	1.0	1.0	1.0	1.0
AKI	185	3.1 (1.9–5.0)	3.2 (2.0–5.1)	3.4 (2.1–5.5)	3.7 (2.2–6.0)	2.8 (1.7–4.8)
**Subgroup analysis**						
No AKI	1531	1.0 (reference)	1.0	1.0	1.0	1.0
AKI stage 1	124	2.7 (1.6–5.0)	2.8 (1.6–5.0)	3.3 (1.8–5.9)	3.6 (2.0–6.6)	3.0 (1.6–5.5)
Aki stage 2 & 3	61	3.8 (1.9–7.5)	3.8 (1.9–7.7)	3.5 (1.7–7.1)	3.8 (1.8–7.8)	2.6 (1.2–5.6)

a. Correction made for: age, gender.

b. Correction made for: age, gender, comorbidities, baseline renal function, immune status, smoking status.

c. Correction made for: age, gender, comorbidities, baseline renal function, immune status, smoking status, medication use

d. Correction made for: age, gender, comorbidities, baseline renal function, immune status, smoking status, medication use, disease severity (MEWS), diagnosis in the emergency department.

**Table 3:** Association between acute kidney injury and in-hospital mortality.

	N	Crude HR(95%CI)	Model 1^a^ HR (95%CI)	Model 2^b^ HR (95%CI)	Model 3^c^ HR (95%CI)	Model 4^d^ HR (95% CI)
No AKI	1531	1.0 (reference)	1.0	1.0	1.0	1.0
AKI	185	4.9 (2.8–8.5)	4.9 (2.9–8.6)	5.0 (2.9–8.7)	5.2 (2.9–9.2)	4.0 (2.2–7.4)
**Subgroup analysis**						
No AKI	1531	1.0 (reference)	1.0	1.0	1.0	1.0
AKI stage 1	124	4.8 (2.5–9.0)	4.8 (2.6–9.1)	4.9 (2.6–9.3)	5.1 (2.7–10.0)	4.2 (2.1–8.3)
Aki stage 2 & 3	61	5.2 (2.3–11.7)	5.2 (2.3–11.8)	5.2 (2.3–11.8)	5.2 (2.3–12.0)	3.7 (1.5–9.0)

a. Correction made for: age, gender.

b. Correction made for: age, gender, comorbidities, baseline renal function, immune status, smoking status.

c. Correction made for: age, gender, comorbidities, baseline renal function, immune status, smoking status, medication use

d. Correction made for: age, gender, comorbidities, baseline renal function, immune status, smoking status, medication use, disease severity (MEWS), diagnosis in the emergency department.

### Long-term renal function in AKI and non-AKI patients

Of the 1716 patients that were included in our analyses, 1467 (85.5%) patients had a preserved renal function and 249 (14.5%) patients ended with an impaired renal function (defined as SCr ≥30% above baseline) up to one year after presentation at the emergency department. The cumulative incidence of renal function decline was 69.8% for the AKI group and 39.3% for the non-AKI group. In our study, we observed an incidence rate for renal function decline of 641 cases per 1000 person-years over 1 year of follow-up in patients surviving an episode of AKI as compared to 212 cases per 1000 person-years in patients who did not have AKI. In addition, patients with an episode of AKI had higher risks of developing renal impairment (adjusted HR 3.3 (95% CI 2.4–4.5) up to one year after initial AKI-episode, even after adjustment for age, gender, comorbidities, immune status, smoking status, medication use, disease severity and diagnosis in the emergency department (Table 4).

**Table 4:** Association between acute kidney injury and renal function decline up to one year after initial acute kidney injury episode.

	N	Incidence rate per 1000 person years	Crude HR (95%CI)	Model 1^a^ HR (95%CI)	Model 2^b^ HR (95%CI)	Model 3^c^ HR (95%CI)	Model 4^d^ HR (95% CI)
No AKI	1531	212	1.0 (reference)	1.0	1.0	1.0	1.0
AKI	185	641	3.5 (2.6–4.7)	3.5 (2.6–4.7)	3.4 (2.6–4.6)	3.4 (2.6–4.5)	3.3 (2.4–4.5)

a. Correction made for: age, gender.

b. Correction made for: age, gender, comorbidities, baseline renal function, immune status, smoking status.

c. Correction made for: age, gender, comorbidities, baseline renal function, immune status, smoking status, medication use.

d. Correction made for: age, gender, comorbidities, baseline renal function, immune status, smoking status, medication use, disease severity in emergency department, diagnosis in the emergency department.

## Discussion

In this study of patients presented to the emergency department for the internal medicine with suspected infection, we found that 10.8% was suffering from an episode of AKI. Importantly, after adjustment for baseline characteristics, comorbidities and diagnosis at emergency department, occurrence of AKI on admission was independently associated with increased 30-days mortality as well as increased risk of renal function decline up to one year after AKI-episode at presentation to the emergency department. To our knowledge, this is the first study to describe the incidence of AKI and associated increased 30-days mortality and renal function decline up to one year after AKI-episode in patients presenting at the emergency department with different infections. Given the high incidence and associated worse outcomes, our study suggest that AKI is a common entity in patients in the emergency department and that awareness as well as early recognition will probably be of great importance and improve patient outcomes.

The epidemiology of AKI in hospitalized and critically ill patients with infection and sepsis has well been described with reported incidence rates that varies between 5–20% among hospitalized patients and 35–50% in critically ill patients [25]. However, studies about incidence rates for AKI and outcomes in patients admitted to the emergency department, which is frequently the first site of care for patients who develop AKI, are scarce and varies due to different patient populations and definitions of AKI used. The observed incidence for AKI in our study is in line with data reported in the study by Scheuermeyer et al. In this retrospective cohort study among 840 unselected patients admitted to the emergency department, they reported an incidence of 10.7% [8]. A study of Tollitt et al. among 17 287 patients with suspected community acquired infection, also showed an incidence rate of 11.9% [9]. Although, both studies included patients with and without baseline SCr, they used the KDIGO criteria for the definition of AKI, consistently with our study. In addition, two other studies found incidence rates of 16 (7) and 34% (8) in patients with (non-severe) community acquired pneumonia and severe pneumonia, respectively, presenting at the emergency department. However, the disparities in incidence rates could be explained by definition used in these studies (RIFLE-criteria) and our study (KDIGO) and severity of illness [12, 26]. Moreover, in the study of Murugan, they used in most of the patients estimated baseline SCr levels, while in our study we included patients with existing baseline SCr values [12].

There is increasing evidence that even a small decrease in renal function in AKI patients is associated with poor short-term and long-term outcomes. This association is extremely high in critically ill patients with infection and sepsis when compared to other causes for AKI [27–29]. In our study, we also found that AKI was associated with a higher 30-days mortality risk and renal function loss up to one year after initial AKI-episode in patients with suspected infection. These findings are consistent with other studies that showed higher mortality in AKI patients, even in non-critical ill patients with infection and in patients that were not admitted to the ICU [12, 30–32]. The study of Murugan et al. and Akram et al. showed that 30-days and 1-year mortality, respectively, were increased in patients with AKI in pneumonia and that the risk increases with increasing levels of renal dysfunction [12, 26]. In our study, we found that 30-days mortality in AKI-patients was 12.4%. In addition, we found an increased risk for mortality, with a HR of 2.8 in AKI patients. Consistently, previous studies have also shown an association between AKI episode and long-term renal dysfunction after hospital admission and discharge, but no studies reported on long-term renal function after AKI in patients at the emergency department, and especially in patients with suspected infection [33].

Severe infection in septic patients is the leading cause of AKI in critically ill patients. AKI in the setting of critically ill patients is associated with ICU admissions, greater hemodynamic complications and adverse outcomes, including cardiovascular events, chronic renal dysfunction, hospital readmission and high mortality [34–37]. Importantly, even studies in patients with early reversal of kidney function after AKI show a higher risk of mortality and progression to chronic kidney disease [38–40]. Although many studies proposed to explore the possible mechanisms between acute renal failure and progression to persistent renal dysfunction in patients with infection or sepsis, exact pathways are still poorly understood. Accumulating evidence in animal studies suggest that persistent renal injury in AKI is the result of an imbalance between pro-inflammatory and anti-inflammatory regulatory mechanisms, resulting in capillary rarefaction and myofibroblast activation with consequently development of fibrosis and scarring in the kidney tissue [37, 41, 42]. Although the kidney has the capacity to recover from acute and limited damage, critical illness, repeated injury, age and comorbidities can contribute to incomplete and maladaptive repair leading to chronic kidney injury and persistent renal dysfunction [35, 36, 41]. Targeted interventions in the early course of AKI, aiming at reducing the transition from AKI to persistent chronic kidney damage, may prevent further damage and improve renal outcomes and reduce mortality rates.

Although there are no targeted therapies for AKI, initial management is crucial and involves recognition, therapy directed at the underlying cause, monitoring of renal function, prevention and adjustment of nephrotoxic medication and RRT if required. Because early AKI recognition might support timely and effective treatment, significant attention has been paid to the importance of early identification of patients with AKI, to limit progression to sustained renal impairment and improve long-term outcomes. However, prompt identification of AKI remains challenging due to a lack of awareness of the importance of early recognition, different care environments where AKI occurs (ICU, hospitalized patients, emergency department, primary care) and diversity of patient populations [43]. Recent studies have reported on the implementation of more specific tools to enhance the awareness and recognition with beneficial impact on long-term outcomes. Especially, the implementation of educational programs for healthcare workers with different backgrounds, electronic alerts for AKI and early nephrology consultation showed a positive effect on outcomes [15, 19, 44, 45]. A recent study of Connell et al. reported a significant improvement in AKI recognition and adjustment of nephrotoxic medication after implementation of a digitally-enabled care pathway for the recognition and management of AKI at the emergency department [46]. In addition, given the association between AKI and long-term renal dysfunction, nephrology consultation can also be of importance for the follow-up of renal function after discharge [44, 47–49]. Interestingly, the development of machine learning models and new biomarkers (e.g. Dickkopf-3) to predict AKI in different patient populations shows promising results, however, the impact of these tools on renal and patient outcomes in the long-term needs to be further evaluated [49, 50].

The current study has several strengths. To the best of our knowledge, this is the first study investigating the incidence, mortality and renal outcomes of AKI in patients with suspected infection presenting at the emergency department with a follow-up to one year. Although several studies reported on 30-days and 1-year outcomes after initial AKI episode at the emergency department, these studies did not include renal specific outcomes [8, 12, 26]. Another strength of our study is that, in contrast to other studies investigating AKI in patients with specific infections at the emergency department, we enrolled patients with different infections. Nevertheless, our study has also several limitations. First, this study is a single center study in an academic center with severely ill patients, which may limits the generalizability to other populations. Secondly, for the baseline SCr levels, we used the most recent SCr values up to 12 months before admission to the emergency department and included only patients with pre-admission baseline SCr from the SPACE-cohort, which may affect overall results by yielding a sicker cohort with predisposing comorbidities compared to healthier patients without these (outpatient) health records. However, we do not think that this has affected the association between AKI and mortality or renal function decline. Moreover, the median period between baseline SCr and presentation at the emergency department was short (28 days (IQR 13–73). Third, the definition of AKI was based only on KDIGO criteria with SCr levels only and did not include urine output. Since creatinine levels can be affected by different conditions (nutrition, infection, muscle mass, age), it could be that classification of AKI based on only SCr may not have captured all cases of AKI. Indeed, a recent study of Kaddourah et al. demonstrated differences in outcomes by using either SCr, urine output or both for the KDIGO definition of AKI [51]. Lastly, we did not perform routine SCr measurements at fixed time-points, therefore AKI episodes might remain undetected.

In conclusion, AKI is a common entity in patients with suspected infection presenting at the emergency department and is associated with 30-days mortality and renal function decline during 1-year follow-up. Our study underscores the importance of enhancing awareness of AKI under treating physicians at the emergency department. More studies are needed to explore better tools for early detection of AKI and improve patient and renal outcomes early intervention.
